# Transcriptome profiling shows gene regulation patterns in ginsenoside pathway in response to methyl jasmonate in *Panax Quinquefolium* adventitious root

**DOI:** 10.1038/srep37263

**Published:** 2016-11-23

**Authors:** Juan Wang, Jinxin Li, Jianli Li, Shujie Liu, Xiaolei Wu, Jing Li, Wenyuan Gao

**Affiliations:** 1 Tianjin Key Laboratory for Modern Drug Delivery and High Efficiency, School of Pharmaceutical Science and Technology, Tianjin University, Tianjin 300072, China; 2 Key Laboratory of Systems Bioengineering, Ministry of Education, Tianjin University, Tianjin 300072, China; 3 Key Laboratory of Industrial Fermentation Microbiology, Ministry of Education, Tianjin University of Science and Technology, Tianjin 300457, China; 4 Tianjin ZhongXin Pharmaceuticals R&D Center, Tianjin 300457, China

## Abstract

Here, we combine elicitors and transcriptomics to investigate the inducible biosynthesis of the ginsenoside from the *Panax quinquefolium*. Treatment of *P. quinquefolium* adventitious root with methyl jasmonate (MJ) results in an increase in ginsenoside content (43.66 mg/g compared to 8.32 mg/g in control group). Therefore, we sequenced the transcriptome of native and MJ treated adventitious root in order to elucidate the key differentially expressed genes (DEGs) in the ginsenoside biosynthetic pathway. Through DEG analysis, we found that 5,759 unigenes were up-regulated and 6,389 unigenes down-regulated in response to MJ treatment. Several defense-related genes (48) were identified, participating in salicylic acid (SA), jasmonic acid (JA), nitric oxide (NO) and abscisic acid (ABA) signal pathway. Additionally, we mapped 72 unigenes to the ginsenoside biosynthetic pathway. Four cytochrome P450s (CYP450) were likely to catalyze hydroxylation at C-16 (c15743_g1, c39772_g1, c55422_g1) and C-30 (c52011_g1) of the triterpene backbone. UDP-xylose synthases (c52571_g3) was selected as the candidate, which was likely to involve in ginsenoside Rb_3_ biosynthesis.

*Panax quinquefolium* L., commonly known as American ginseng, belongs to the *Araliaceae* family and has gained tremendous global trade and recognition as a health food supplement. The dried root powder of this plant has been used extensively for its antitumor, anti-stress, anti-ageing, anti-fatigue, cardioprotective and hepatoprotective properties^1^,^2^. Ginsenoside are secondary metabolites of the *P. quinquefolium* and its major pharmacologically active components. Ginsenosides are divided into protopanaxadiol, protopanaxatriols and oleanolic acid based on their structure.

A 5-year planting cycle is required before the mature roots of *P. quinquefolium* can be harvested. Ginsenoside production in *P. quinquefolium* is generally low and difficult to synthesize chemically, limiting the utility of ginsenosides. Alternatively, enhancing ginsenoside production through genetic manipulation of its secondary metabolic pathways is a potential strategy for improving yield. However, this requires extensive knowledge of the ginsenoside biosynthetic pathway and, along with its medical importance, has led to extensive research in the area. Studies have resulted in the identification of ginsenoside biosynthetic enzymes, including 3-hydroxy-3-methylglutaryl CoA reductase (HMGR), geranyl diphosphate synthase (GPS), farnesyl diphosphate synthase (FPS), squalene synthase (SS)^3^,^4^, squalene epoxidase (SE), dammarenediol synthase (DS) and β-amyrin synthase (β-AS)^5^,^6^. It is known that ginsenoside biosynthesis is achieved mainly through three reaction steps of 2, 3-oxidosqualene: cyclization, hydroxylation and glucosidation^7^. However, despite these advances, further elucidation of the ginsenoside biosynthetic pathways has been slowed by the limited sequence information available for cytochrome P450 (CYP450) (CYP716A53v2, CYP716A47, CYP716A52v2)^8^,^9^ and glycosyltransferase (GT) (UGT74AE2, UGT94Q2, UGT71A27, UGTPg1, UGTPg100, UGTPg101)^10^,^11^,^12^.

Our laboratory previously demonstrated that ginsenoside production is enhanced by the addition of methyl jasmonate (MJ) into *Panax ginseng* adventitious roots^13^. The elicitors can be recognized by plant receptors which are located on the surface of the plasma membrane or endomembrane. The receptors are activated, and then in turn activate their effectors, such as ion channels, GTP binding proteins (G-proteins), and protein kinases and oxidative burst^14^. Activated effectors can promote the synthesis of signaling molecules, such as salicylic acid (SA), jasmonic acid (JA), nitric oxide (NO), abscisic acid (ABA) and so on, which transfer the elicitor signals to defense genes that have been induced by elicitor treatment, and further amplify the elicitor signal to the biosynthesis of secondary metabolites^15^.

In order to elucidate the genes involved in the ginsenoside biosynthetic pathway, we sequenced, then compared two sets of transcriptome profiles derived from MJ treated and untreated *P. quinquefolium* adventitious roots. In addition, signaling molecules and defense genes in response to MJ were also studied. Our results provide a foundation for genetically constructing the ginsenoside biosynthetic pathways, which will in turn aid the study of its regulation and metabolic engineering of ginsenoside compounds.

## Results and Discussion

### Effects of MJ on active compound content

Treatment of adventitious root cultures with MJ stimulated ginsenoside accumulation (43.66 mg/g) compared with the control group (8.32 mg/g), decreasing root biomass and polysaccharide content (Fig. S1a–d). The biomass showed a negative correlation with electrical conductivity (EC) (Fig. S1b). Finally, these results led to higher ginsenoside productivity (105.74 mg/l) compared to the control group (30.19 mg/l) (Fig. S1f).

Treatment of *P. quinquefolium* adventitious root cultures with MJ resuled in an increase in ginsenoside content. MJ, a derivative of jasmonic acid, is an effective elicitor that is involved in plant defense response pathways and triggers plant metabolite biosynthesis. Therefore, MJ has been used for inducing metabolite production in plant cell cultures. MJ treatment is known to activate proteinase inhibitor genes in plants, which is likely to explain the observed decrease in biomass^16^.

### MJ-induced NO, SA, JA and ABA accumulation

**Figure 1:**
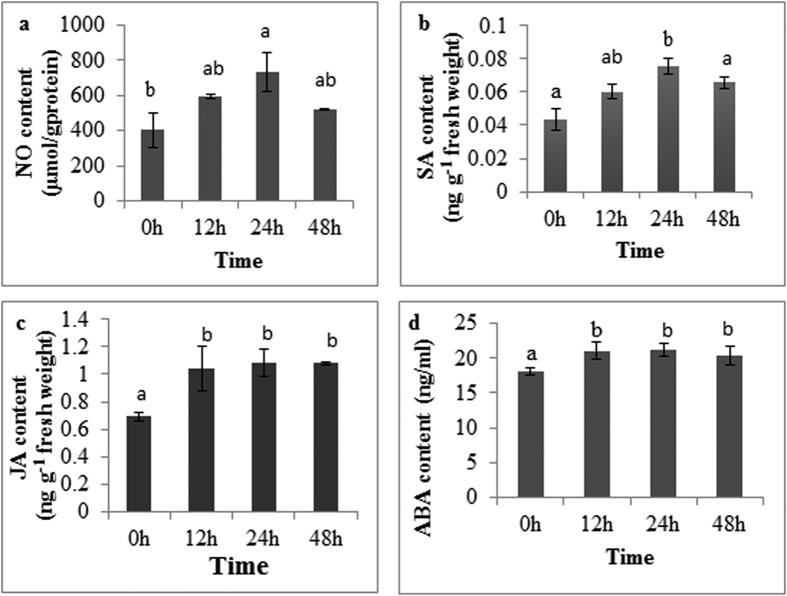
Accumulation of signal molecules: NO (**a**), SA (**b**), JA (**c**), ABA (**d**) in adventitious roots of *P. quinquefolium* that were affected by MJ.

The results of this study showed that MJ can induce NO, SA, JA and ABA accumulation in *P. quinquefolium* adventitious roots. As shown in Fig. 1, increase of NO, SA, JA and ABA were observed, reaching the highest level (732.44 μmol·gprotein^−1^, 0.08 ng·g^−1^, 1.08 ng·g^−1^ and 21.11 ng·mL^−1^) at 24 h, respectively.

NO is a key signal molecule in plant that induces a defense response to elicitors. It has been reported that NO can participate in the secondary metabolite accumulation such as ginsenosides^17^, taxanes^18^ and other bioactive compounds. Hu *et al*.^17^ found that NO was required for oligogalacturonic acid-induced saponin synthesis in cell cultures of *P. ginseng*. Furthermore, elicitors also induce the accumulation of SA and JA in plant cell. Xu *et al*.^19^ found that SA and JA have synergistic effects on regulating elicitor-induced puerarin accumulation in cell culture. ABA acts as an important signal molecule to regulate biosynthesis of secondary metabolites in some plant cell. ABA can stimulate production of indole alkaloids in *C. roseus* cell culture^20^ and taxol production in *Taxus spp* cell culture^21^. In this work, MJ induced the accumulation of signal molecules (NO, SA, JA and ABA) and enhanced the ginsenosides contents.

### Functional annotation and gene ontology classification

RNA samples were extracted from control and MJ treated *P. quinquefolium* adventitious roots. Illumina RNA sequencing technology was used to sequence the whole transcriptome of *P. quinquefolium*. Unigenes with sequence orientation were aligned against public databases such as the Nr, SwissProt, Nt, Pfam, COG (Clusters of Orthologous Groups of proteins), GO (Gene Ontology) and KO (KEGG Ortholog).

**Figure 2:**
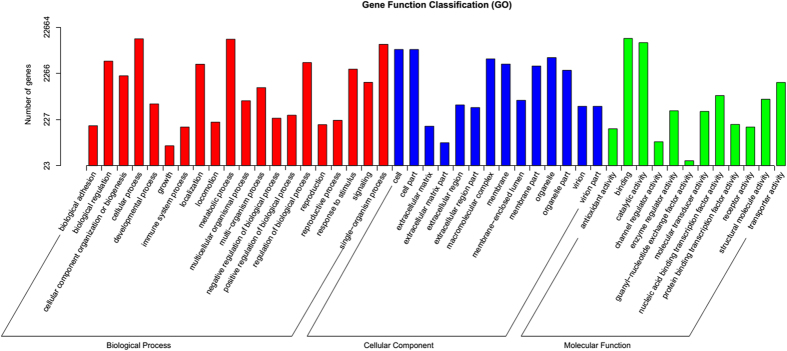
Histogram of gene ontology classification.

Unigenes with Nr annotation were further annotated and classified under GO. GO is an international standardized gene functional classification system. Among the GO classifications, assignments to the biological process class ranked highest (286,854), followed by cellular component (82,491) and molecular function (56,853). Within the biological process category, the majority of the GO terms were assigned to cellular and metabolic processes. Within the cellular components category, transcripts assigned to cell and cell parts were the most common. For molecular function, the assignments were mostly binding and catalytic activity (Fig. 2). Transcripts related to GO term binding were most abundant in the molecular function category.

**Figure 3:**
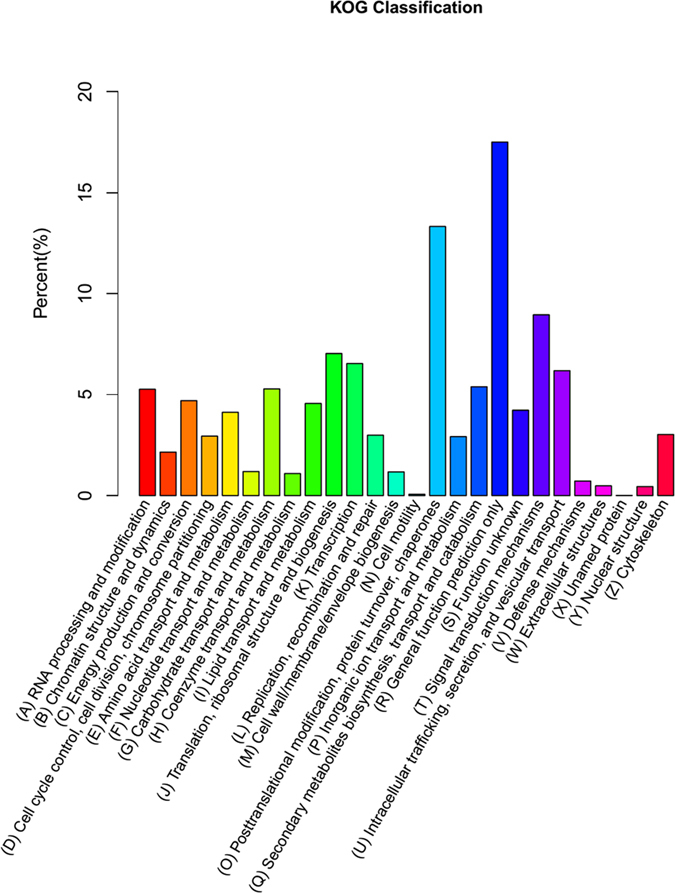
Histogram of unigene KOG classification.

COG is a classification system based on orthologous genes. Orthologous genes have the same function and a common ancestor. We annotated 12,354 unigenes to 26 groups using the COG database (Fig. 3). The largest number (1,929) of the annotated unigenes fell within the general functional prediction only (R), while the fewest number of unigenes (1) were annotated as an unnamed protein (X). Additional assignments included 593 unigenes within the secondary metabolites biosynthesis, transport and catabolism (Q) category and 79 as defense mechanism unigenes (V) (Fig. 3).

For medicinal plants, RNA-seq has been used to identify genes that are directly or indirectly involved in the biosynthetic pathways of target bioactive compounds^22^. Transcriptome analysis from only one plant organ or tissue does not provide a full transcript catalogue, even though it can serve numerous specific genetic and breeding objectives. In our current study, we generated a total of 201 million high-quality reads from control and MJ treated adventitious roots, which is significantly more than the previously reported data of 454 ESTs^3^. Functional genomics studies require highly reliable reference sequences. Therefore, the transcript library we assembled here has significant implications for functional genomic studies on *P. quinquefolium* due to the high sequencing depth. Transcripts related to GO term binding were most abundant in the molecular function category. This is in agreement with the previously reported GO annotation of *P. ginseng* adventitious roots^23^.

### Differentially expressed unigenes analysis

Unigene expression was calculated using a reads per kb per million reads (RPKM) method. A total of 12,148 DEGs were identified in the two experiments (control and MJ) including 5,759 up-regulated and 6,389 down-regulated genes.

GO functional analysis was also integrated with the clustering analysis of expression patterns. Within the DEGs group, metabolic process, cell and cell part genes were found to be abundant (Fig. S2a). Within the up-regulated DEGs, GO terms related to the biosynthetic process as well as intracellular and single-organism metabolic processes were significantly enriched (Fig. S2b). By contrast, genes primarily down-regulated after induction were related to binding and heterocyclic compound binding (Fig. S2c).

Approximately 29% of the DEGs were categorized as genes responsive to stimuli and stress (Fig. S2a), suggesting that the expression of a large number of genes may be altered in response to an external stimulus^24^.

### Pathway analysis

**Figure 4:**
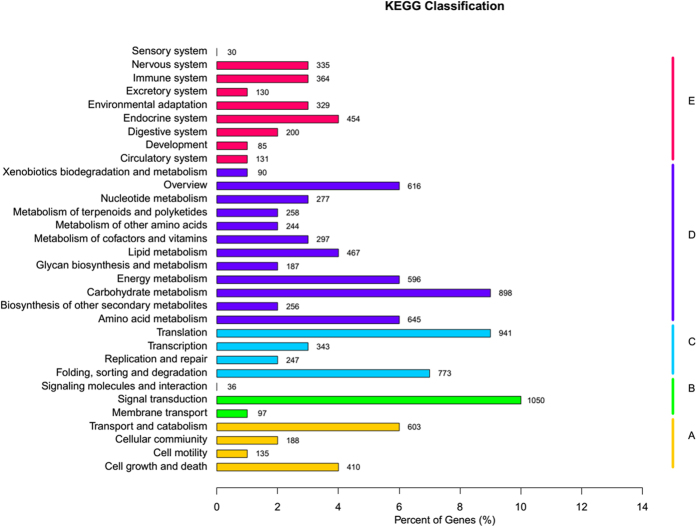
Functional classification and pathway assignment of unigenes by KEGG.

Pathway-based analysis provides information on biological functions and the synthesis of secondary metabolites, particularly at the molecular level. For MJ treated *P. quinquefolium* adventitious roots, a total of 16,371 transcripts were assigned to 32 KEGG (Kyoto Encyclopedia of Genes and Genomes) pathways (Fig. 4). As shown in Fig. 4, the majority of KEGG assigned transcripts were involved in signal transduction (1,050). A large pool of transcripts fell within the area of carbohydrate metabolism biosynthesis (898). Additional transcripts were mapped to the area of lipid metabolism biosynthesis (467) and metabolism of terpenoids and polyketides biosynthesis (258).

Of the DEGs that were significantly up- or down-regulated, only 12,148 were KO assigned. DEGs involving the spliceosome (118 DEGs), ribosome (186 DEGs) and RNA transport (110 DEGs) were significantly enriched pathways (qvalue < 0.1) (Fig. S3a). In addition, DEGs pathways that were significantly up-regulated included the ribosome (178 DEGs), amino acid biosynthesis (107 DEGs), fatty acid biosynthesis (22 DEGs), carbon metabolism (101 DEGs), biotin metabolism (11 DEGs), citrate cycle (TCA cycle) (30 DEGs), unsaturated fatty acid biosynthesis (27 DEGs), 2-oxocarboxylic acid metabolism (25 DEGs), fatty acid metabolism (44 DEGs), stilbenoid, diarylheptanoid and gingerol biosynthesis (10 DEGs), arachidonic acid metabolism (10 DEGs), phenylalanine, tyrosine and tryptophan biosynthesis (20 DEGs), pyruvate metabolism (45 DEGs) and terpenoid backbone biosynthesis (28 DEGs) (qvalue < 0.1) (Fig. S3b).

Pathway assignment for all transcripts was performed based on the KEGG database. In the case of *Panax*, well-represented pathways included amino acid metabolism, carbohydrate metabolism, lipid metabolism^3^,^25^ and energy metabolism^26^. In this study, MJ were chosen as exogenous precursors to increase ginsenoside production in *P. quiquefolium* adventitious roots. Most primary metabolic processes such as the citrate cycle, carbohydrate metabolism, and amino acid metabolism were significantly up-regulated pathways among the DGEs. Primary metabolism is essential for plant growth, plant development and plant reproduction. In cell suspension cultures, primary metabolism is essential for plant cells to propagate in liquid media^22^.

### Analysis of defense genes

**Figure 5:**
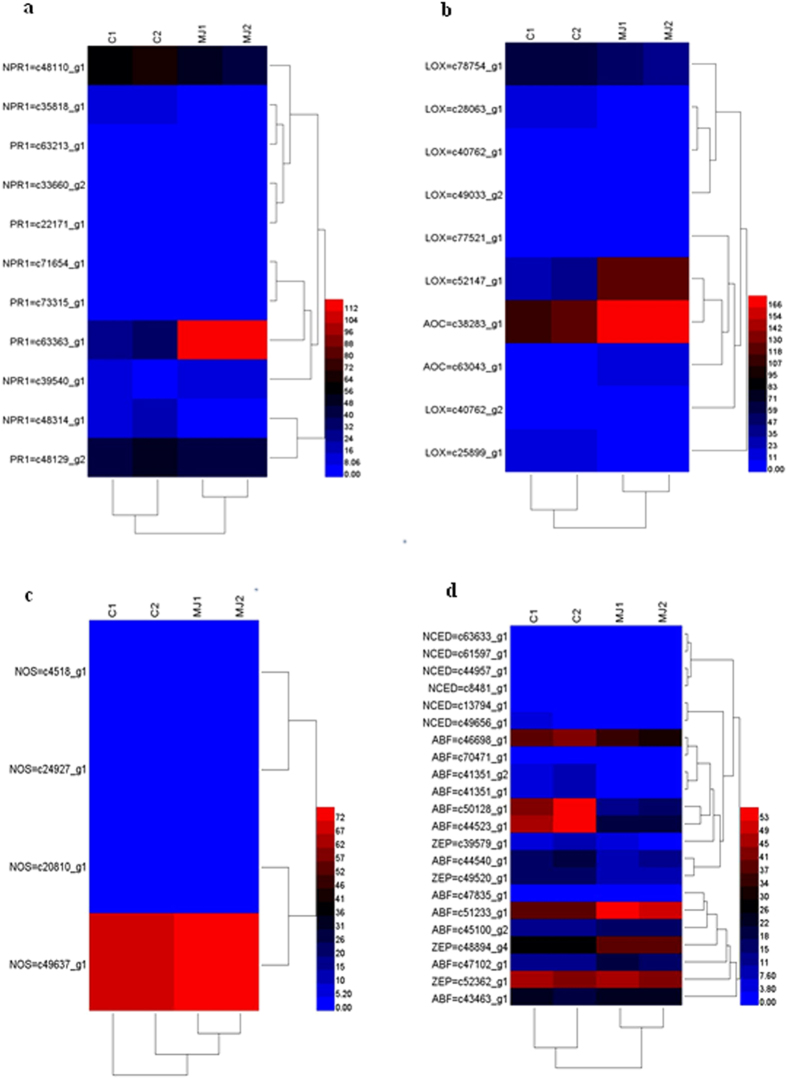
RNAseq-based transcript profiling of defense genes. (**a**) Genes encoding known proteins involved in SA signal pathway. (**b**) Genes encoding known proteins involved in JA signal pathway. (**c**) Genes encoding known proteins involved in NO signal pathway. (**d**) Genes encoding known proteins involved in ABA signal pathway.

Across all samples, several defense-related genes were identified, participating in SA, JA, NO and ABA signal pathway. pathogenesis-related protein 1 (PR1), allene oxide cyclase (AOC), 9-cis-epoxycarotenoid dioxygenase (NCED), ABA responsive element binding factor (ABF) and zeaxanthin epoxidase (ZEP) showed a pronounced up-regulation by MJ elicitation (Fig. 5).

NPR1 acts as a receptor of the SA signal and then activities PR genes expression, including PR1^27^. On the other hand, the JA signaling pathway is positively regulated by the nuclearlocalized helix-loop-helix-leucine zipper-type transcription factor MYC2 and induces plant defense related proteins, such as AOC and LOX^28^. Recent studies showed that ABA involved in a complicated network of synergistic and antagonistic interactions with other phytohormones. Signaling related genes that are modified by ABA include NCED, ABF, ZEP and sucrose nonfermenting 1-related protein kinase 2^29^. Besides, we have not identify NO-related defense genes that up-regulated significantly. In this study, the expression of PR1, AOC, NCED, ABF and ZEP up-regulated significantly, which indicated that the signal molecules, generated after MJ treatment, enhanced ginsenosides content by regulating defense genes, consisting with the results of previous studies. Cerato-platanin triggers SA-signaling pathways, as revealed by the expression of PR genes and induced the biosynthesis of camalexin^30^.

### Analysis of genes involved in ginsenoside biosynthesis

Using our RNA-seq data, we inspected the expression of genes from the upstream triterpenoid precursor biosynthetic pathways, named the cytosolic mevalonic acid (MVA) pathway and the plastidial 1-deoxy-D-xylulose-5-phosphate pathway.

In the transcriptome data, we found that 72 unigenes were mapped to the ginsenoside biosynthesis pathway. The most abundant unigenes (13) were assigned as HMGR and DXP. Within the putative ginsenoside biosynthetic genes, 29 unigenes were up-regulated while 7 were down-regulated. Among the DEGs, putative HMGR (5), 1-deoxy-D-xylulose-5-phosphate synthase (DXP) (4), isopentenyl diphosphate isomerase (IPPI) (1), geranyl diphosphate synthase (GPS) (6), FPS (1), SE (2), β-amyrin synthase (β-AS) (2), P450 (4) and GT (4) were significantly up-regulated (Table 1).

We observed an up-regulation of genes involved in mono-, sesqui- and tri-terpenoid metabolism. These data are in agreement with previous studies that reported transcriptional up-regulation of the precursor pathways that likely increase synthesis of terpenoid natural products^31^. Mechanistically, MJ binds to membrane receptors and activates G-proteins to trigger phospholipase A (PLA). Subsequently, PLA activates α-linolenic acid and endo-methyl jasmonate. Endogenous MJ regulates the HMGR pathway^32^ and down-stream genes to produce the mono-, di-, sesqui-, and tri-terpenoid genes^23^. From our investigation, we also found that the most abundant up-regulated unigenes were assigned to HMGR and GPS in response to MJ. Regulation of the cycloartenol synthase (CAS) and lupeol synthase (LS) genes, leading to production of phytosterols and lupeol, did not significantly change. These data suggested that treatment of adventitious roots with MJ resulted in the attenuation of competitive pathways and eventually diversion of the metabolic flux to the production of the desired ginsenosides.

### Analysis of putative genes involved in the late steps of ginsenoside biosynthesis

As one of the best-characterized protein families, CYP450s are known to catalyze the oxidation function of carbon-carbon bonds as well as alkyl hydroxylation and hydroxyl oxidation reactions^33^. Our RNA-seq data revealed 7 CYP450s (c52011_g1, c48642_g1, c15743_g1, c39772_g1, c38567_g1, c35627_g1, c55422_g1) that likely involved in ginsenoside biosynthesis. GTs are another large multigene family in plants. In this study, a total of 5 GTs unique sequences (c52571_g3, c45579_g2, c47755_g1, c39632_g1, c51194_g1) were found and likely to be involved in ginsenoside biosynthesis. Thus, 7 CYP450s and 5 GTs were selected.

**Figure 6:**
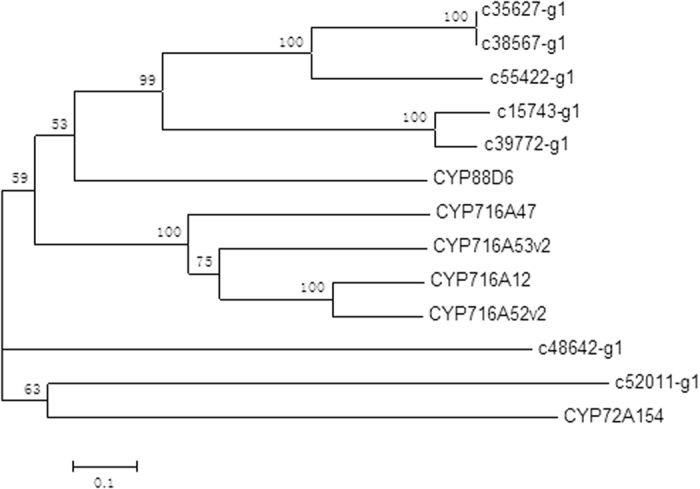
Phylogenetic analysis of P450s from *P. quinquefolium* adventitious root and other P450s involved in triterpene saponin biosynthesis.

The phylogenetic relationship between the 7 fulllength CYP450s of *P. quinquefolium* adventitious root and characterized CYP450s from other plants was depicted in Fig. 6. It is noteworthy that c15743_g1, c39772_g1, c38567_g1, c35627_g1, c55422_g1 were phylogenetically close to CYP88D6, a β-amyrin 11-oxidase from *G. uralensis*^34^. Phylogenetic analysis also found that the obtained full-length of c52011_g1 was close to CYP72A154, a β-amyrin 30-oxidase from *G. uralensis*^35^.

**Figure 7:**
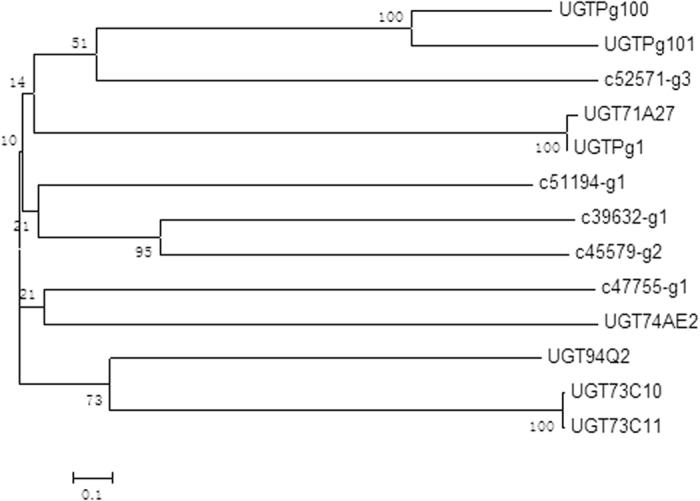
Phylogenetic analysis of GTs from *P. quinquefolium* adventitious root and other GTs involved in triterpene saponin biosynthesis.

Phylogenetic analysis showed the relationship of *P. quinquefolium* adventitious root GT sequences to other functionally characterized members of plant GT families (Fig. 7). Among them, alpha-1,3-glucosyltransferase (c47755_g1) and UDP-xylose synthases (c52571_g3) were regarded as a lead candidate GTs responsible for triterpene saponin biosynthesis, because of its close relation to triterpene glucosyltransferases UGTPg100, UGTPg101, UGT74AE2. Hence, there were 6 candidate CYP450 unigenes and 2 candidate GT unigenes.

**Figure 8:**
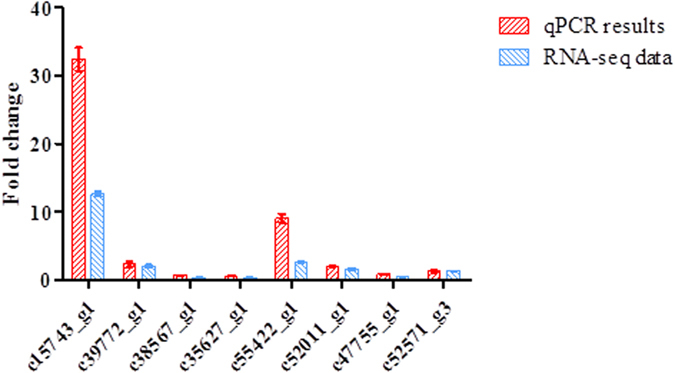
Expression pattern validation of selected unigenes by qRT-PCR.

Quantitative PCR analysis was performed on 8 selected CYP450 and GT genes putatively involved in the ginsenoside biosynthesis of *P. quinquefolium*. The qPCR results of 8 selected genes showed general agreement with their transcript abundance changes as determined by RNA-seq (Fig. 8). Four P450 genes (c15743_g1, c52011_g1, c39772_g1, c55422_g1), and one UDP-xylose synthases (c52571_g3) showed a significant up-regulation in response to MJ.

CYP450 and GT enzymes are critical for the downstream metabolism of ginsenosides to produce protopanaxadiol and protopanaxatriol. CYP450 monooxygenases play a key role in terpenoid biosynthesis, with such activity almost invariably required for further transformation of olefinic intermediates^31^. So far, the P450 compendium that can oxidize the dammarane and amyrin backbone is expanded, now covering six positions on the triterpene backbone and including C-6 (CYP716A53v2)^8^, C-11 (CYP88D6)^34^, C-12 (CYP716A47)^8^, C-16 (CYP716Y1)^36^, C-28 (CYP716A52v2, CYP716A12)^9^ and C-30 (CYP72A154)^35^. In this study, phylogenetic and PCR analysis found that the c15743_g1, c39772_g1, c55422_g1 were close to CYP88D6 and c52011_g1 was close to CYP72A154.

GT glycosylation of natural compounds is an important mechanism for detoxification of a wide variety of exogenous compounds^37^. In general, glycosylation is the last step in the biosynthesis of secondary metabolites. Recently, identified eight GTs (UGT73C11, UGT73C10, UGT74AE2, UGT94Q2, UGT71A27, UGTPg1, UGTPg100, UGTPg101) involved in the later steps of ginsenoside biosynthesis in the closely related species *P. ginseng*^10^,^11^,^12^.

In the studies on transcriptome analysis of *Panax notoginseng*, 350 and 342 unigenes were predicted to encode CYP450s and GTs, respectively^38^. However, the gene function of CYP P450s and GTs had not been predicted through phylogenetic analysis or qPCR. Results of *Panax ginseng* adventitious roots showed that, putative uncharacterized CYPs (PG027814, PG024387, PG024073, PG019557, PG002087, PG005498, PG000598, PG001995) and GTs (PG010742, PG002718, PG025219, PG002650, PG000627) were highly co-expressed with ginsenoside pathway related transcripts and transcription factors^39^. In this study, UDP-xylose synthases (c52571_g3) were regarded as a lead candidate GT responsible for Rb3 (1,6-xylosyltransferase) biosynthesis, because of its close relation to 1,6-glucosyltransferases UGTPg100 and UGTPg101. Here, we found that four CYP450s (c15743_g1, c39772_g1, c55422_g1, c52011_g1) and one UDP-xylose synthases (c52571_g3), which were likely to be involved in ginsenoside biosynthesis.

### Effects of MJ on expression of functional genes

**Figure 9:**
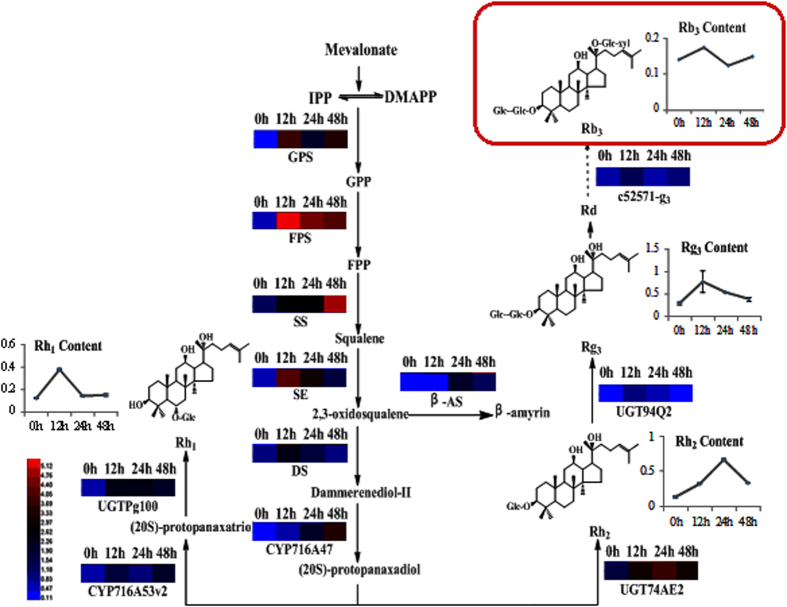
Putative ginsenoside biosynthesis and expression of functional genes.

Genes expression level (GPS, FPS, SS, SE, β-AS, DS, CYP716A47, CYP716A53v2, UGT74AE2, UGT94Q2, UGTPg100 and c52571-g3) in adventitious roots after MJ treatment in different time (0 h, 12 h, 24 h and 48 h) were studied. The expression levels of functional genes were up-regulated compared with untreated group. In particularly, the expression levels of UGT74AE2, UGT94Q2 and UGTPg100 that generate Rh_2_, Rg_3_ and Rh_1_ respectively, reaching its peak at 24 h, 12 h and 12 h respectively, which consisted with production of monomer ginsenoside Rh_2_, Rg_3_, and Rh_1_. Besides, the expression level of c52571-g3, a candidate gene responsible for Rb_3_, was also up-regulated (Fig. 9).

In *Artemisia annua*, MJ induced artemisinin biosynthesis by up-regulating the expression of the genes involved in artemisinin biosynthesis^40^. This study showed that exposure to MJ in adventitious roots of *P. quinquefolium* enhanced the production of ginsenosides through regulated the expression of functional genes involved in triterpene biosynthesis.

In summary, we report here a validated large-scale transcriptome data set of *P. quinquefolium* adventitious roots. This study provides an important resource for understanding the formation and accumulation of secondary metabolites, paving the way for industrialization of ginsenosides.

## Methods

### Plant material

The 5-year-old roots of *P. quinquefolium* were obtained from Zuo Jia Institute, Chinese Academy of Agricultural Sciences, Jilin, China. The details of adventitious roots, medium and culture conditions have been described previously^41^.

### MJ treatment

Adventitious roots (10 g·l^−1^) were inoculated into 5 l balloon-type bubble bioreactors (BTBBs) containing 3 l 3/4 strength Murashige and Skoog (MS) (Murashige and Skoog, 1962) liquid medium supplemented with 3.0 mg·l^−1^ IBA, 1.0 mg·l^−1^ NAA and 4% sucrose. No elicitors were added to the control cultures. After 28 days of culture, MJ (5.0 mg·l^−1^) were added into the medium and the samples were then allowed to continue culturing for 12 additional days. The growth ratio, electrical conductivity (EC), total saponins and polysaccharide content were determined on day 28, 32, 36 and 40. Each experiment was repeated at least three times.

After pre-cultivation for four weeks, 5.0 mg L^−1^ MJ was added to the *P. quinquefolium* adventitious roots medium. The roots were taken at 0, 12, 24 or 48 h to determine signal molecule (SA, JA, NO and ABA), ginsenoside content and expression level of functional genes. Culture conditions were the same as above. Each experiment was repeated at least three times.

### Library preparation and sequencing

Total RNA of control and MJ-treated adventitious roots for 12 h were isolated using the Plant RNA KitII(OMEGA, USA). RNA integrity was assessed using the RNA Nano 6000 Assay Kit of the Agilent Bioanalyzer 2100 system (Agilent Technologies, CA, USA). A total amount of 3 μg RNA per sample was used as input material for the RNA sample preparations. Sequencing libraries were generated using NEBNext^®^ Ultra™ RNA Library Prep Kit for Illumina^®^ (NEB, USA) following manufacturer’s recommendations. Index codes were added to each sample to identify attributes for each sequence. The clustering of the index-coded samples was performed on a cBot Cluster Generation System using TruSeq PE Cluster Kit v3-cBot-HS (Illumia) according to the manufacturer’s instructions. After cluster generation, the library preparations were sequenced on an Illumina Hiseq 2500 platform and paired-end reads were generated.

### Transcript assembly and annotation

The left files (read1 files) from all libraries/samples were pooled and labeled as left.fq file. Similarly, the right files (read2 files) were pooled and labeled as right.fq file. Transcriptome assembly was accomplished based on the left.fq and right.fq files using Trinity^42^ with min_kmer_cov set to 2 and all other parameters left at default settings.

Gene function was annotated based on the following databases: Nr (NCBI non-redundant protein sequences); Nt (NCBI non-redundant nucleotide sequences); Pfam (Protein family); COG (Clusters of Orthologous Groups of proteins); Swiss-Prot (A manually annotated and reviewed protein sequence database); KO (Kyoto Encyclopedia of Genes and Genomes (KEGG) Ortholog database); GO (Gene Ontology).

### Differential expression analysis

Differential expression analysis of two conditions was performed using the DESeq R package (1.10.1). DESeq provide statistical routines for determining differential expression in digital gene expression data using a model based on the negative binomial distribution. The resulting values were adjusted using the Benjaminiand Hochberg’s approach for controlling the false discovery rate. Genes with an adjusted P-value < 0.05 found by DESeq were assigned as differentially expressed.

### GO and KEGG enrichment analysis

GO enrichment analysis of the differentially expressed genes (DEGs) was implemented by the GOseq R packages, basing on Wallenius non-central hyper-geometric distribution^43^, which can adjust for gene length bias in DEGs. We used KOBAS^44^ software to test the statistical enrichment of DGEs in KEGG pathways.

### Quantitation of SA, JA, NO and ABA

0.25 g of adventitious roots (control group and MJ group) were ground into powder using a mortar and pestle chilled with liquid nitrogen. Extraction and analyses of SA and JA were performed as described previously^45^. The extracts of NO and ABA were prepared by homogenizing 0.2 g of adventitious roots (control group and MJ group) in a mortar on ice, using 1.0 mL distilled water. The contents of NO and ABA were measured using commercially available kits (Nanjing Jiancheng Bioengineering Research Institute, Nanjing, China) according to the manufacturer’s instructions.

### Quantitative PCR

For each qRT-PCR reaction, 200 ng of total RNA was used for first strand cDNA synthesis. First-strand cDNAs were used as a template for RT-PCR reactions, which were performed as follows: 94 °C for 2 min, then 35 cycles of 94 °C for 30 s, 57 °C for 1 min, and 72 °C for 50 s; with a final 2 min extension at 72 °C. We used the ABI7500 for quantitative PCR reactions and the relative standard curve method was adopted to analyze the relative expression of genes. The PCR products were determined by agarose gel (2%) electrophoresis. The size of the fragments was estimated using a 100-bp ladder (CWBIO, China) as a size marker. All experiments were performed in triplicate. The primers of genes used in RT-PCR are shown in Table S1, Table S2.

### HPLC analysis

Samples were analyzed using an Agilent HPLC system containing a surveyor autosampler. The details of the analytical procedures have been previously described^41^,^46^.

### Phylogenetic analysis

Probable entire amino acid sequences of CYP7450s and UGTs were taken from the GenBank database (http://www.ncbi.nlm.nih.gov) and evolutionary distances were computed using the Poisson correction method, and a Neighbor-Joining (NJ) tree was constructed with MEGA4. The indicated scale represents 0.1 amino acid substitutions per site. Bootstrap values obtained after 1000 replications are given on the branches.

## Additional Information

**How to cite this article**: Wang, J. *et al*. Transcriptome profiling shows gene regulation patterns in ginsenoside pathway in response to methyl jasmonate in *Panax Quinquefolium* adventitious root. *Sci. Rep.* **6**, 37263; doi: 10.1038/srep37263 (2016).

**Publisher's note:** Springer Nature remains neutral with regard to jurisdictional claims in published maps and institutional affiliations.

## Supplementary Material

Supplementary Information

## Figures and Tables

**Table 1:** Unigenes potentially related to ginsenoside biosynthesis in *P. quinquefolium*.

Enzyme name	Abbreviations	KO	Total unigene	Up-regulated	Down-regulated
hydroxymethylglutaryl-CoA synthase	HMGR	K01641	3	1	0
hydroxymethylglutaryl-CoA reductase (NADPH)		K00021	10	4	0
mevalonate kinase	MVA	K00869	1	0	0
phosphomevalonate kinase		K00938	6	0	1
diphosphomevalonate decarboxylase		K01597	1	0	0
1-deoxy-D-xylulose-5-phosphate synthase	DXP	K01662	11	2	2
1-deoxy-D-xylulose-5-phosphate reductoisomerase		K00099	2	2	0
isopentenyl-diphosphate delta-isomerase	ippi	K01823	1	1	0
geranylgeranyl reductase	GPS	K10960	2	2	0
geranylgeranyl diphosphate synthase, type II		K13789	6	3	1
geranyl diphosphate synthase		K14066	1	1	0
farnesol kinase	FPS	K15892	1	1	0
farnesol dehydrogenase		K15891	1	0	1
farnesyl-diphosphate farnesyltransferase		K00801	1	0	0
farnesyl diphosphate synthase		K00787	2	0	0
squalene monooxygenase	SE	K00511	3	2	0
cycloartenol synthase	CAS	K01853	3	0	0
β-amyrin synthase	β-AS	K15813	4	2	0
lupeol synthase 1	LS	K15816	1	0	0
cytochrome P450	CYP450	K07409	7	4	2
		K09832			
		K09588			
		K12639			
dolichyl-diphosphooligosaccharide-protein glycosyltransferase	GT	K07151	5	4	0
