# Supplementary material for: Transcriptome profiling shows gene regulation patterns in ginsenoside pathway in response to methyl jasmonate in Panax Quinquefolium adventitious root
Source: Sci Rep. 2016 Nov 23;6:37263. doi: 10.1038/srep37263 (PMC5120341; doi:10.1038/srep37263)
Supplement: Supplementary Information [file srep37263-s1.pdf]

Transcriptome profiling shows gene regulation patterns in ginsenoside pathway in response to methyl jasmonate in *Panax Quinquefolium* adventitious root

Juan Wang[1,2*#], Jinxin Li[1,2#], Jianli Li[3], Shujie Liu[3], Xiaolei Wu[4], Jing Li[1,2], Wenyuan Gao[1,2*]

Figure legends:

Figure S1 Effects of MJ on growth and active components of *P. quinquefolium* adventitious roots.

Figure S2 Histogram of GO classification of DEGs.

Figure S3 Scatterplot of KEGG enrichment of DEGs. Rich factor: the proportion of DEG in the corresponding pathway.

Table legends:

Table S1 Primers used for qRT-PCR analysis of selected ginsenoside biosynthesis related genes

Table S2 Primers used for qRT-PCR analysis of ginsenoside biosynthesis related genes

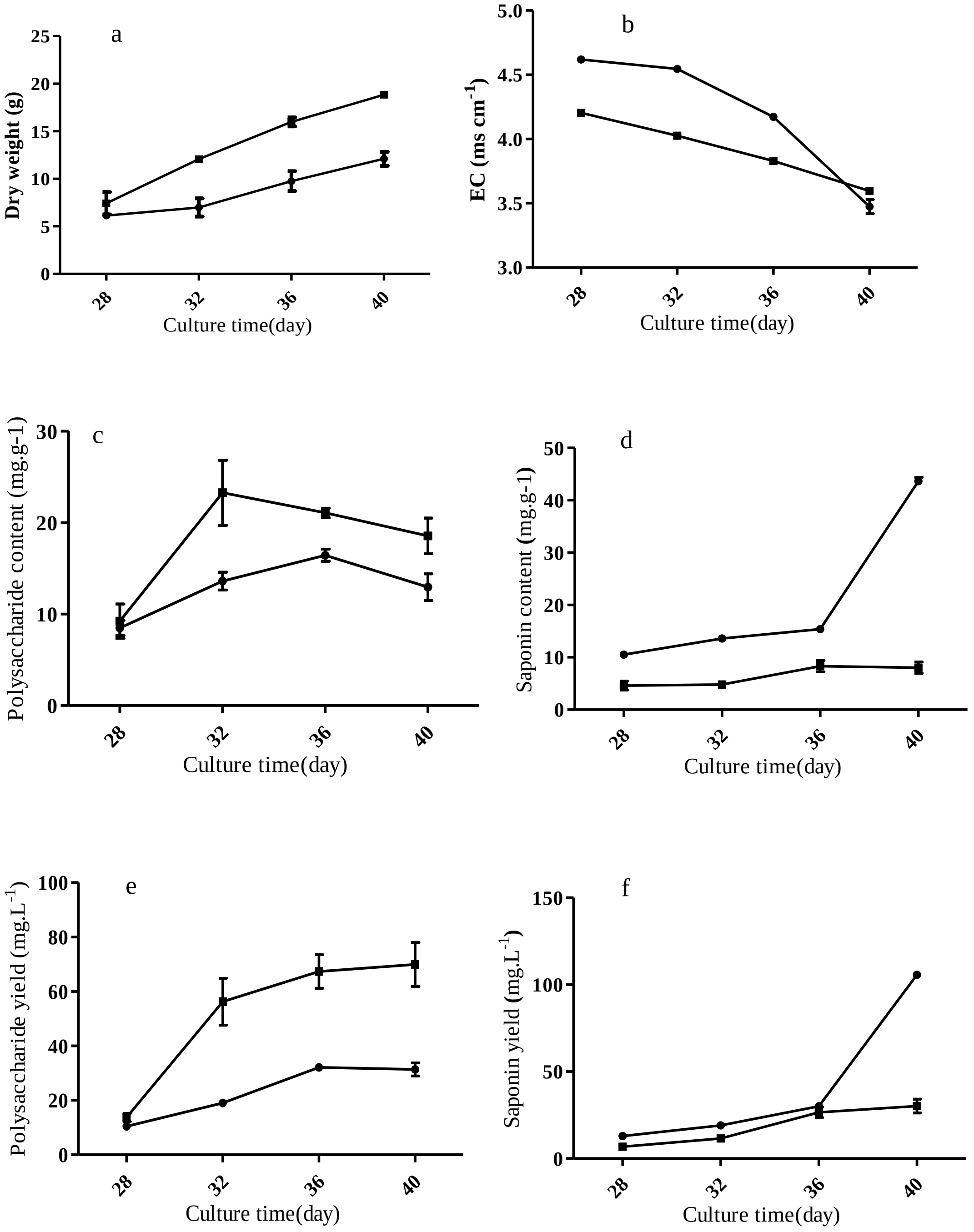

Figure S1 Effects of MJ on growth and active components of *P. quinquefolium* adventitious roots. a: Effects on dry weight; b: Effects on EC; c: Effects on polysaccharide content; d: Effects on saponin content; e: Effects on polysaccharide yield; f: Effects on saponin yield. Symbols: (■) Control group; (●) Elicitor group, adding MJ (5.0 mg/l) on 28th day.

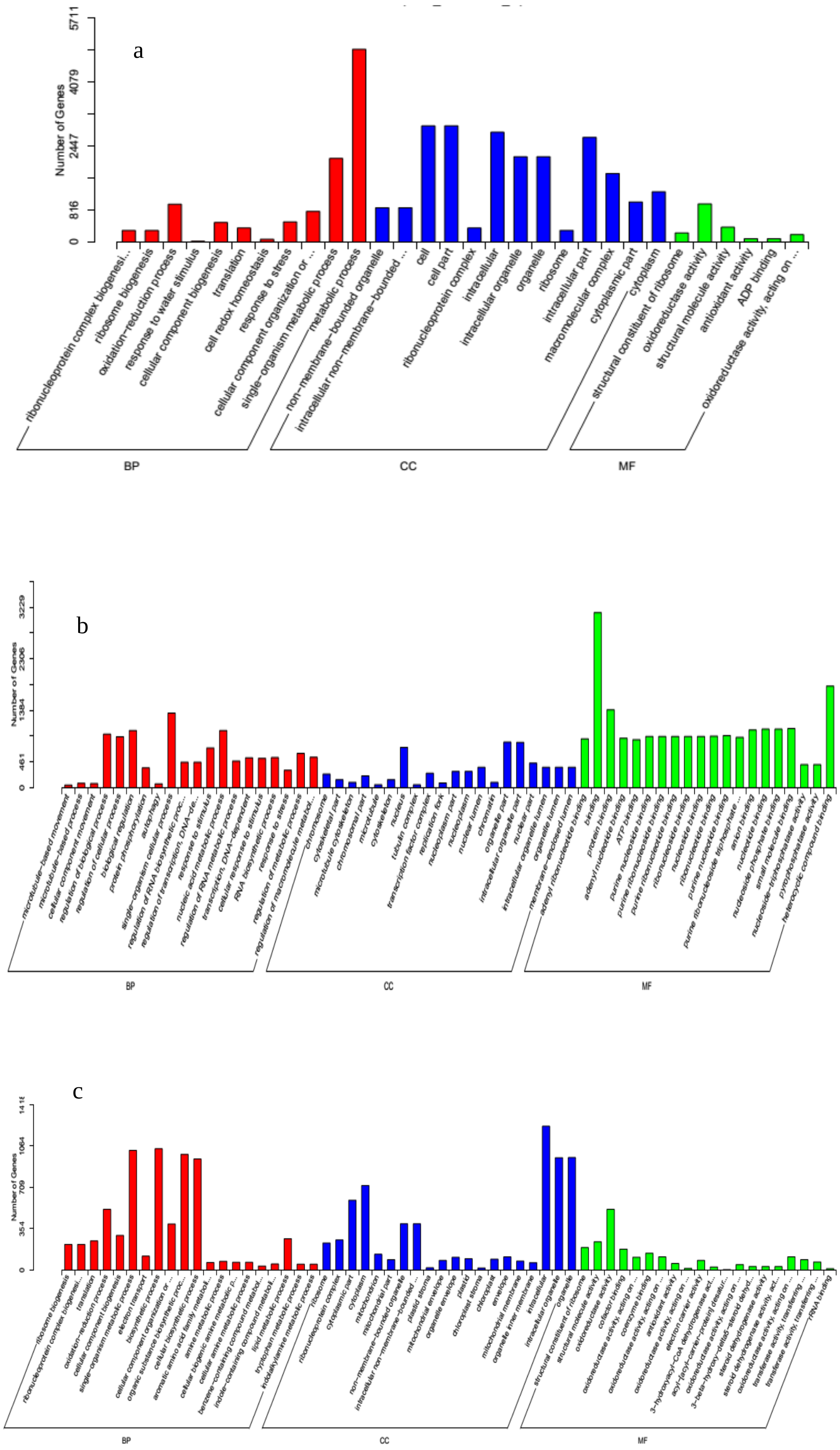
a
Number of Genes
0
816
2447
4079
5711
ribonucleoprotein complex biogenesi...
ribosome biogenesis
oxidation−reduction process
response to water stimulus
cellular component biogenesis
translation
cell redox homeostasis
response to stress
cellular component organization or ...
single−organism metabolic process
metabolic process
non−membrane−bounded organelle
intracellular non−membrane−bounded ...
cell
cell part
ribonucleoprotein complex
intracellular
intracellular organelle
organelle
ribosome
intracellular part
macromolecular complex
cytoplasmic part
cytoplasm
structural constituent of ribosome
oxidoreductase activity
structural molecule activity
antioxidant activity
ADP binding
oxidoreductase activity, acting on ...
BP
CC
MF
b
Number of Genes
0
461
1384
2306
3229
BP
CC
MF
c
Number of Genes
0
354
709
1064
BP
CC
MF

Figure S2 Histogram of GO classification of DEGs. a: DEGs between MJ and control; b: up-regulated genes between MJ and control; c: down-regulated genes between MJ and control.

a

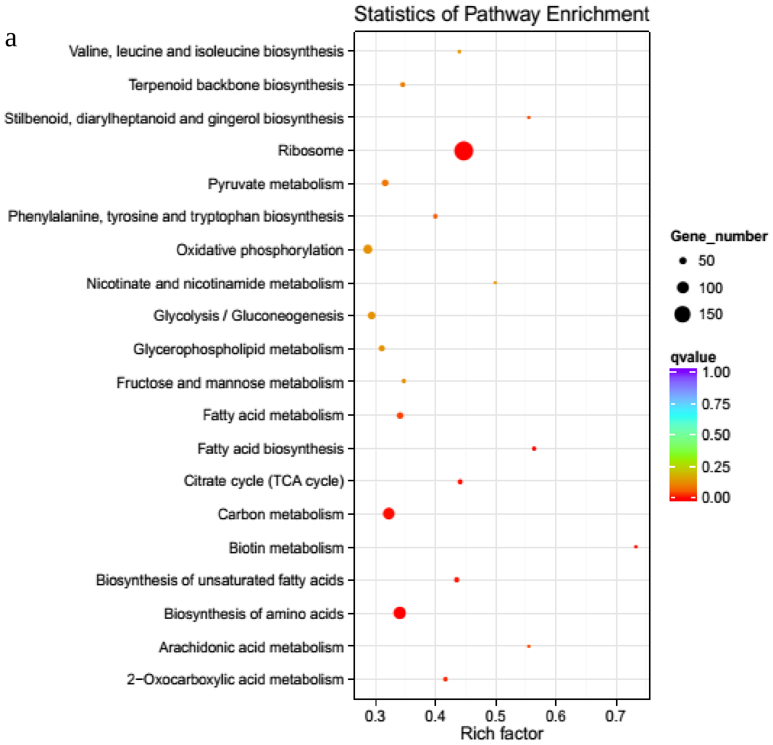
Statistics of Pathway Enrichment
Valine, leucine and isoleucine biosynthesis
Terpenoid backbone biosynthesis
Stilbenoid, diarylheptanoid and gingerol biosynthesis
Ribosome
Pyruvate metabolism
Phenylalanine, tyrosine and tryptophan biosynthesis
Oxidative phosphorylation
Nicotinate and nicotinamide metabolism
Glycolysis / Gluconeogenesis
Glycerophospholipid metabolism
Fructose and mannose metabolism
Fatty acid metabolism
Fatty acid biosynthesis
Citrate cycle (TCA cycle)
Carbon metabolism
Biotin metabolism
Biosynthesis of unsaturated fatty acids
Biosynthesis of amino acids
Arachidonic acid metabolism
2−Oxocarboxylic acid metabolism
0.3
0.4
0.5
0.6
0.7
Rich factor
Gene_number
50
100
150
qvalue
1.00
0.75
0.50
0.25
0.00

b

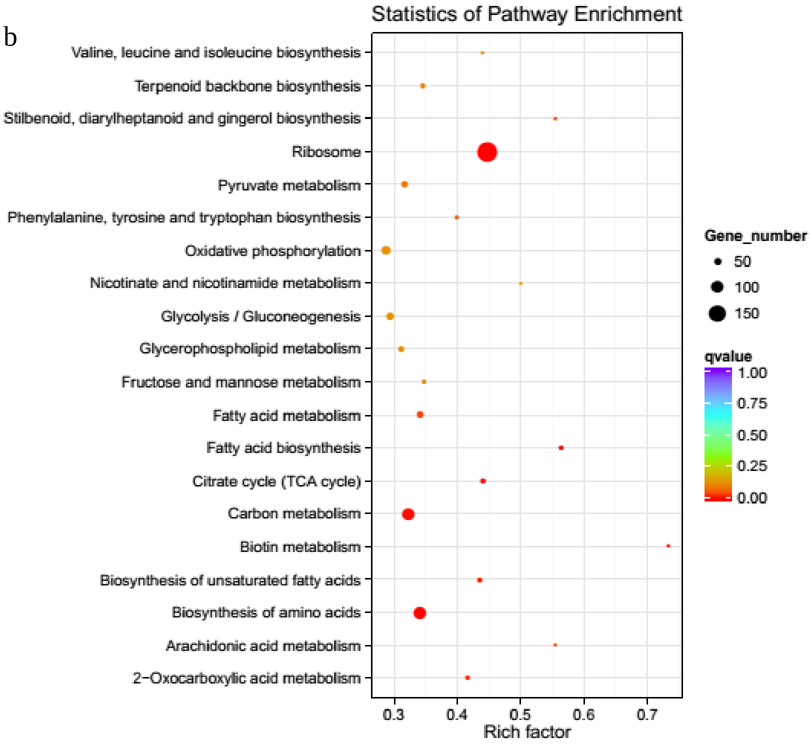
Statistics of Pathway Enrichment
Valine, leucine and isoleucine biosynthesis
Terpenoid backbone biosynthesis
Stilbenoid, diarylheptanoid and gingerol biosynthesis
Ribosome
Pyruvate metabolism
Phenylalanine, tyrosine and tryptophan biosynthesis
Oxidative phosphorylation
Nicotinate and nicotinamide metabolism
Glycolysis / Gluconeogenesis
Glycerophospholipid metabolism
Fructose and mannose metabolism
Fatty acid metabolism
Fatty acid biosynthesis
Citrate cycle (TCA cycle)
Carbon metabolism
Biotin metabolism
Biosynthesis of unsaturated fatty acids
Biosynthesis of amino acids
Arachidonic acid metabolism
2−Oxocarboxylic acid metabolism
0.3
0.4
0.5
0.6
0.7
Rich factor
Gene_number
50
100
150
qvalue
1.00
0.75
0.50
0.25
0.00

c

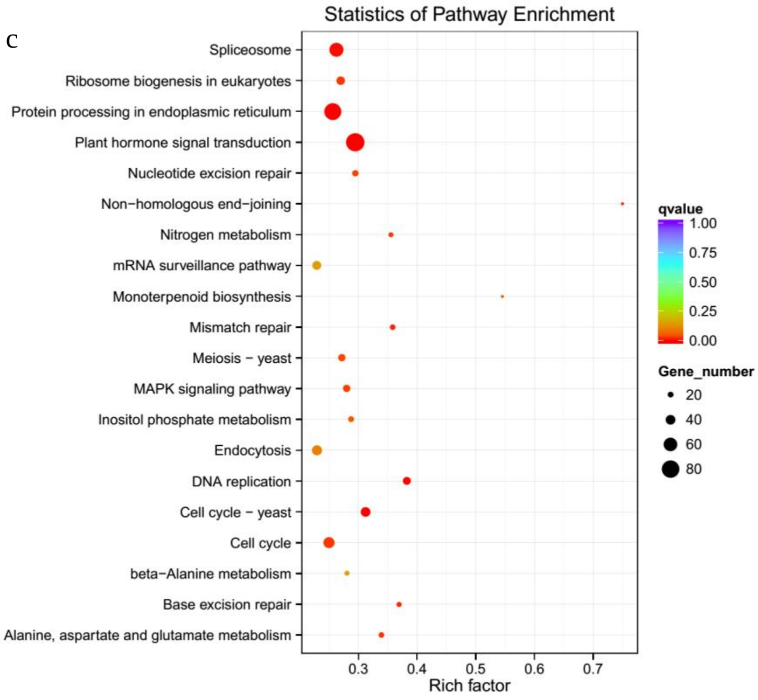

Figure S3 Scatterplot of KEGG enrichment of DEGs. Rich factor: the proportion of DEG in the corresponding pathway. a: KEGG enrichment of DEGs between MJ and control; b: KEGG enrichment of up-regulated genes between MJ and control; c: KEGG enrichment of down-regulated genes between MJ and control.

Table S1 Primers used for qRT-PCR analysis of selected ginsenoside biosynthesis related genes

Gene Name	Primer sequence (5′-3′)
c52011_g1	For: GCAGGAGCAATGGATACAT<br>Rev: GTGGAATGGATAATGGACCT
c15743_g1	For: GAAGCAACTATTGAGCATCG<br>Rev: AACCAACTCCGCCTACATC
c39772_g1	For: AGACTCCTCTCGCTTTGGCT<br>Rev: CACATCGGTCATTGCTCTC
c38567_g1	For: AGAGTTTGTGATACCAGCAGG<br>Rev: TCAGCACCAGGACATAGACG
c35627_g1	For: ATCTCGGTGATGGACTCGT<br>Rev: GGTGCCAGGAACACATAGTG
c55422_g1	For: TGGAAAGTTCTACCCGTCA<br>Rev: GCCATAGGTTGCTCATCATT
c52571_g3	For: GCATCAACATCAAGCACG<br>Rev: CGAGAAGTGAATGAGACGC
c47755_g1	For: TGGTTCAGCAAATACGGG<br>Rev: AGACGACTCTTCCAAAGCC

Table S2 Primers used for qRT-PCR analysis of ginsenoside biosynthesis related genes

Gene Name	Primer sequence(5’-3’)
Actin	For: CAG AAG AGC ACC CTG TTC TTT<br>Rev: ATA AAT GGG GAC TGT GTG GCT
GPS	For: GTC AGA ATT GAT ATT CTT GCC CGC CC<br>Rev: ATG TCT CGC ACG TGT GTC TTC T
FPS	For: CTG AAA TCC GAG CTA CTC AAC GA<br>Rev: GCC ATT CAA TGC ACC AAC CA
SS	For: ATG GGA AGT TTG GGG GCA ATT CT<br>Rev: GTT CTC ACT GTT TGT TCA GTA GTA GGT T
SE	For: AGC AGC AGT TGA CAA AGG<br>Rev: GCC ACA TTC GTT TTG GTG AAG G
DS	For: CGG AAA CGT GTT TGG TTG CC<br>Rev: CAA ACA ACA CCT ATT TCC GAT T
β-AS	For: TAT CCT GGA CAC CGA AAG AAG G<br>Rev: CTC CAC TTA TTT CCT GTT GGG G
CYP716A47	For: ATG GTG TTG TTT TTC TCC CTA TCT

	Rev: TTA ATT GTG GGG ATG TAG ATG AAT
CYP716A53v2	For: ATG GAT CTC TTT ATC TCATCT CAA<br>Rev: TTA AAG CGT ACA AGG TGA TAG ACG
UGT74AE2	For: ATG CTG AGC AAA ACT CAC ATT A<br>Rev: AAC TCC CAT ATA AGC CTG CAT
UGT94Q2	For: GGT AGA ATC AGT ATA GCG TTG C<br>Rev: TCT GAG TGG GAG CAT GAG ATG GGA A
UGTPg100	For: ATG GAT CTC TTT ATC TCA TCT CAA<br>Rev: TTA AAG CGT ACA AGG TGA TAG ACG
